# Mapping the potential suitable habitats for *Hyalomma rufipes* (Acari: Ixodidae) in Africa and Western Eurasia

**DOI:** 10.1371/journal.pntd.0012923

**Published:** 2025-03-18

**Authors:** Ruobing Zhou, Hein Sprong, Qiyong Liu, Thomas Krafft, Agustin Estrada-Peña

**Affiliations:** 1 Department of Health, Ethics & Society, CAPHRI Care and Public Health Research Institute, Faculty of Health, Medicine and Life Sciences, Maastricht University, Maastricht, the Netherlands; 2 Centre for Zoonoses and Environmental Microbiology, National Institute for Public Health and the Environment (RIVM), Bilthoven, the Netherlands; 3 National Key Laboratory of Intelligent Tracking and Forecasting for Infectious Diseases, National Institute for Communicable Disease Control and Prevention, Chinese Center for Disease Control and Prevention, Beijing, China; 4 Department of Animal Health, Faculty of Veterinary Medicine, University of Zaragoza, Spain; 5 Instituto Agroalimentario de Aragón, Zaragoza, Spain (retired),; 6 Ministry of Human Health, Madrid, Spain; Huazhong University of Science and Technology Tongji Medical College, CHINA

## Abstract

Crimean-Congo hemorrhagic fever is a broadly distributed tick-borne disease and is caused by the arthropod-borne Crimean-Congo hemorrhagic fever virus (CCHFV). *Hyalomma* ticks have been associated with the circulation of the virus in natural foci and in laboratory experiments. One of the main species, *Hyalomma rufipes*, is originally distributed in Africa. However, anthropogenic activities, bird migration, and domestic animal movement, could break the natural barriers that prevent its spread out of its natural area of colonization. This study explored the potential suitable areas for *H. rufipes* in Africa, Southern Europe and Central Asia using an environmental niche model. Explanatory variables based on climate were generated by harmonic regression of long-term climate; records of *H. rufipes* were obtained from public databases or provided by other scientists and researchers. The model indicated that areas likely to support permanent populations of *H. rufipes* are distributed across Southern Africa, Northern Africa, Southern Europe, the Arabian Peninsula, and the Caucasus. Data on migratory birds infested with *H. rufipes* further supports the need for surveillance activities in these regions to monitor and manage both the vectors and the pathogens they carry.

## Author summary

*Hyalomma rufipes* is one of the main vectors of Crimean-Congo hemorrhagic fever virus (CCHFV). We mapped the potential suitable area of *H. rufipes* in partial Africa and Eurasia. The model indicated that Areas with higher possibility to be suitable for *H. rufipes* include Northern and Southern Africa, the Western and Southern Iberian Peninsula, the Arabian Peninsula, southern coastal areas of France and Italy, Cyprus, Türkiye, parts of Greece, the Caucasus region, and Iran and Afghanistan. By integrating data on migratory birds infested with *H. rufipes* with our findings, it becomes clear that the Mediterranean region warrants closer monitoring for tick surveillance and prevention. This study enhances the understanding of the potential distribution of CCHF vector and provides insights for vector and CCHF surveillance.

## Introduction

Crimean Congo hemorrhagic fever (CCHF) is a broadly distributed tick-borne disease in the Old World, with a case fatality rate ranging from 5 to 12% [1, 2]. In recent decades, the emergence or re-emergence of human clinical cases has been reported in several countries across Africa, the Middle East, Asia, and Southern Europe [3,4]. CCHF is caused by the arthropod-borne CCHF virus (CCHFV) (species *Orthonairovirus haemorrhagiae*, genus *Orthonairovirus*, family *Nairoviridae*) [4], primarily transmitted to humans through the bite or handling of an infected tick. It can also spread through direct contact with the blood or tissues of viremic animals and human cases. *Hyalomma* ticks, most notably *Hyalomma marginatum, Hyalomma turanicum, Hyalomma asiaticum,* and *Hyalomma rufipes,* serve as vectors and reservoirs for CCHFV through vertical transmission, possessing the necessary competence for its maintenance and transmission [5,6].

A recent classification system has been proposed to assess country-specific CCHFV surveillance based on case reports, vector presence, and existing surveillance activities. This system includes five levels, ranging from Level One, where human CCHF cases are reported annually and the virus is endemic, to Level Five, where no information is available [7,8]. Given the absence of licensed vaccines or therapeutics for CCHF, One Health surveillance is particularly valuable in high-risk countries. It integrates awareness, preparedness, and preventive measures, which are essential for minimizing pathogen transmission and reducing disease incidence and mortality [9,10]. Evidence suggests that the geographic distribution of *Hyalomma* species indicates potential CCHFV circulation and associated human CCHF risk [11].

Studies have modelled potential suitable habitats for one CCHF vector, *H. marginatum* [12,13]. Nevertheless, the current distribution data for this tick species does not fully account for the distribution of human cases or positive serological findings in domestic animals, suggesting that other tick species may also need to be considered [14]. For example, *H. rufipes* is known to thrive better in places with higher accumulated temperature than *H. marginatum* [6,15]. Given that CCHFV has been detected in *H. rufipes* collected from birds or domestic animals [16,17], this species play a role in viral transmission [6]. *Hyalomma rufipes* is a two-host tick, larvae and nymphs feed on small mammals, ground-dwelling birds, and reptiles. Adult ticks feed on ungulates such as goats, camels, horses, and cattle, occasionally humans [18,19]. *Hyalomma rufipes* is a widespread species that has been found in Southern Europe (probably as an invader, these ticks were likely introduced through animal dispersal or human activities but have not yet established permanent, reproducing populations.), North and sub-Saharan Africa, and Central Asia, extending into China [20–28]. Recently, records of its presence have become more common in Central and Northern Europe [23,25]. Studies conducted in Africa and Europe indicate that the survival of *H. rufipes* survival is limited by the combined effects of accumulated temperature and the amount of water in the air. The former regulates life cycle processes such as molting, oviposition, and incubation, while the latter influences mortality rates[15]. Building upon these findings, this study aims to investigate the potential (environmental) suitable range for *H. rufipes.*

Environmental Niche Modeling (ENM) is widely applied in ecology, providing essential information for various fields, including conservation, biological invasions, and the geography of disease transmission risk [29–31]. ENM associates biodiversity data with environmental variables to estimate potential suitable areas for species. Among the various algorithms behind ENM, Maximum Entropy Modeling (MaxEnt) has gained significant popularity in research over the past few decades [32,33], due to its user-friendly interface and strong performance. On the other hand, it has been demonstrated that explanatory climate variables obtained by harmonic regression from long series of climate data supersede the unreliable use of blindly selected variables; reports of ENM for various organisms commonly use a set of pre-tailored climate variables that are selected according to statistical criteria ignoring its contribution to the biology of the organism to be modeled [15,34–38]. Fourier-derived variables are of ecological relevance to species, rather than being limited by data availability [15].

This study focuses on the detection of the potential suitable climate range for *H. rufipes* (thus ignoring spatial accessibility) using climate variables related to temperature and vapor pressure deficit. We used a large dataset of records of *H. rufipes* assembled from various sources. The main purpose of this study is to identify sites that could support the presence of *H. rufipes* and to provide surveillance recommendations for certain areas.

## Methods

### Climate variables

A set of environmental variables was utilized based on their ecological relevance to *H. rufipes* and utilized as independent variables in the model. The generation of these variables involved several steps: Monthly datasets of mean temperature and mean vapor pressure deficit at a 30-arc-second resolution from 1990 to 2018 were obtained from the CHELSA dataset (version 2.1, https://chelsa-climate.org/timeseries/, accessed on October 15, 2023). We selected a target territory delineated within a rectangular boundary extending from the Atlantic Ocean in the west to 73°E in the east, and from 51°N in the north to South Africa in the south. The average monthly temperature and vapor pressure deficit over the 29-year period for that territory were calculated using the ‘terra’ package [39] in R (version 4.4.0) [40]. The ‘TSA’ package [41] was then employed to derive harmonic regression coefficients. These coefficients conform an equation, in which the variable “time” is included as a fraction of the year to generate values at, e.g., daily or weekly intervals [37]. Running iteratively the equation, we obtained daily data for average temperature and vapor pressure deficit over the 29 years. The ‘changepoint’ package [42] was used to define seasons based on changes and trends of the average temperature [15]. We selected eight descriptive climate variables from daily data based on their ecological meaning and demonstrated utility in previous studies. These variables include: annual accumulated temperature above 0°C, accumulated temperature above 0°C in spring, accumulated temperature above 0°C in summer, amplitude of temperature above 0°C in winter, amplitude of vapor pressure deficit (VPD) in winter, sum of VPD in winter, sum of VPD in autumn, and annual sum of VPD. The threshold of 0°C was chosen to maintain consistency with Estrada (2023), which demonstrated that these variables are relevant to the ecology of *H. rufipes*. To note that the meaning of season (e.g., spring) is not calendar-derived, but supported by the change of slope of temperature, as captured for every single pixel of the complete target territory [15].

### Tick presence records

**Fig 1:**
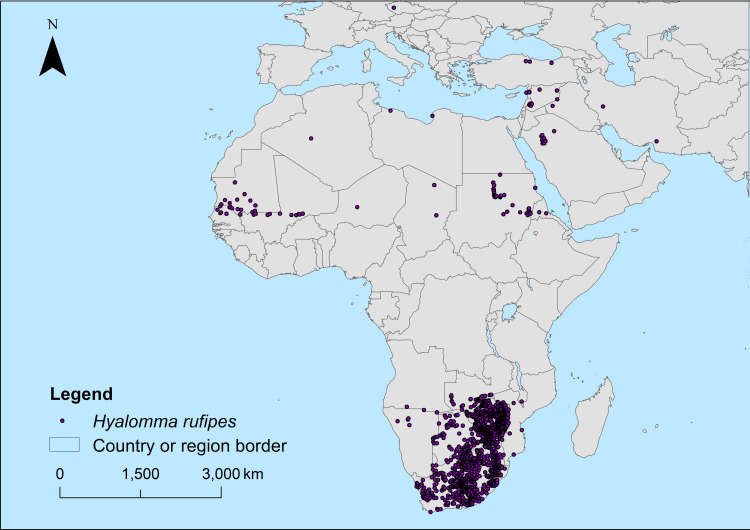
*H. rufipes* presence sites used in model establishment (data was collected between 1990 and 2018, S1 Table). Base layers from (https://hub.arcgis.com/datasets/esri::world-countries-generalized/explore), Terms of use (https://www.esri.com/content/dam/esrisites/en-us/media/legal/product-specific-terms-of-use/e300.pdf).

Records of *H. rufipes* were obtained from multiple sources: (1) Global Biodiversity Information Facility [43–48]; and (2) collaborations by authors of studies on the topic and scientists specialized in tick research (S1 Table). Ticks were identified in these studies using either morphological (e.g., the morphology of spiracular plate and the setae around it) or molecular (sequence alignment) identification methods. To ensure spatial-temporal consistency with climate variables, only records collected between 1990 and 2018 were retained with duplicates removed. Final dataset consists of 1,430 collection sites (Fig 1 and S1 Table). The coordinates of the presence sites were recorded in a dedicated table. Specimens collected from museum collections were excluded from the analysis to ensure that the data reflected relevant field observations. Areas near the equator experience minimal temperature fluctuations throughout the year, which diminishes the effectiveness of the detection of the seasons, that relies on temperature variation. To avoid these issues, records of *H. rufipes* within 15° north and south of the equator were excluded from the analysis. The remaining data were then randomly divided into three subsets using the ‘dplyr’ package [49] in R: 70% for training, 25% for testing, and 5% for evaluation. All data processing steps were conducted in R. It has been reported that some migratory birds carrying *H. rufipes* were captured in the Northern Hemisphere, and their coordinates recorded. This dataset was compiled from both published sources and data provided by coauthors (S2 Table).

### Accessible area and study region

An accessible area with a 10 km radius was defined around each presence site to facilitate model calibration [33,50,51]. We first trained all candidate models within the accessible area, assessed and selected the ones with better performances (further details regarding the assessment are provided in the following sections), then transferred to the entire study region. The environmental variables were then separately masked to fit within these defined geographic boundaries using R.

### Environmental niche modeling

The ENM was built using the MaxEnt algorithm in the *‘*kuenm’ package in R [31]. Candidate models were developed using training dataset, testing dataset, eight descriptive climate variables, along with 19 regularization multipliers (RM, 0.1-1, in steps of 0.1 and 1—10 in steps of 1), with the feature classes including linear, quadratic, and a combination of linear and quadratic [33]. With these varied model configurations, a total of 57 candidate models were constructed (S3 Table). Model performance was assessed using two main criteria: (1) statistical significance, which included partial Receiver Operating Characteristic (pROC) analysis with 100 iterations and 50% of the data for bootstrapping, as well as the omission rate (E = 5%); and (2) model complexity, evaluated by the corrected Akaike Information Criterion (AICc) [52–54]. Models with omission rates lower than 5% were prioritized. Among these, models with delta AICc values less than 2 were selected. Selected models and corresponding parameters were recorded fitting the final model with these parameters and the same dataset of tick presence records. Final models fitting process involved 20 cross-validation replicates and cloglog output, with no variable extrapolation. The models were then transferred from the accessible area to the study region. The evaluation of the final models included calculating the partial ROC and omission rate (5%) using an independent presence dataset. Finally, the results were converted to binary format based on a 10% training presence threshold. Results displays were achieved by ArcMap (version 10.5) [55].

## Results

### Dataset, Environmental variables and parameter selection

Performance of the modeling approaches was explored with respect to the evaluation criteria separately. Out of 57 candidate models, 11 models were statistically significant. All meet omission rate criteria of 5%, 5 with delta AICc value lower than 2. Parameter settings and evaluation results were listed in Table 1. 5 models were transferred to study area and evaluated by independent dataset (Table 2).

**Table 1:** Performance of candidate models meeting evaluation criteria.

Model	RM	Feature	P-value of pROC	Omission rate at 5%	Delta AICc
Model1	7	linear	0.017	0.045	0.000
Model2	7	quadratic	0.017	0.045	0.638
Model3	6	quadratic	0.050	0.047	0.982
Model4	8	linear and quadratic	0.010	0.045	1.291
Model5	7	linear and quadratic	0.007	0.045	1.498

**Table 2:** Final model evaluations for five candidate models (with 20 replicates).

Model	Mean AUC ratio	P-value of pROC	Omission rate at 5%
Model1	1.035	0.000	0.042
Model2	1.051	0.000	0.014
Model3	1.058	0.000	0.000
Model4	1.034	0.000	0.042
Model5	1.036	0.000	0.028

### Transfer to study region

**Fig 2:**
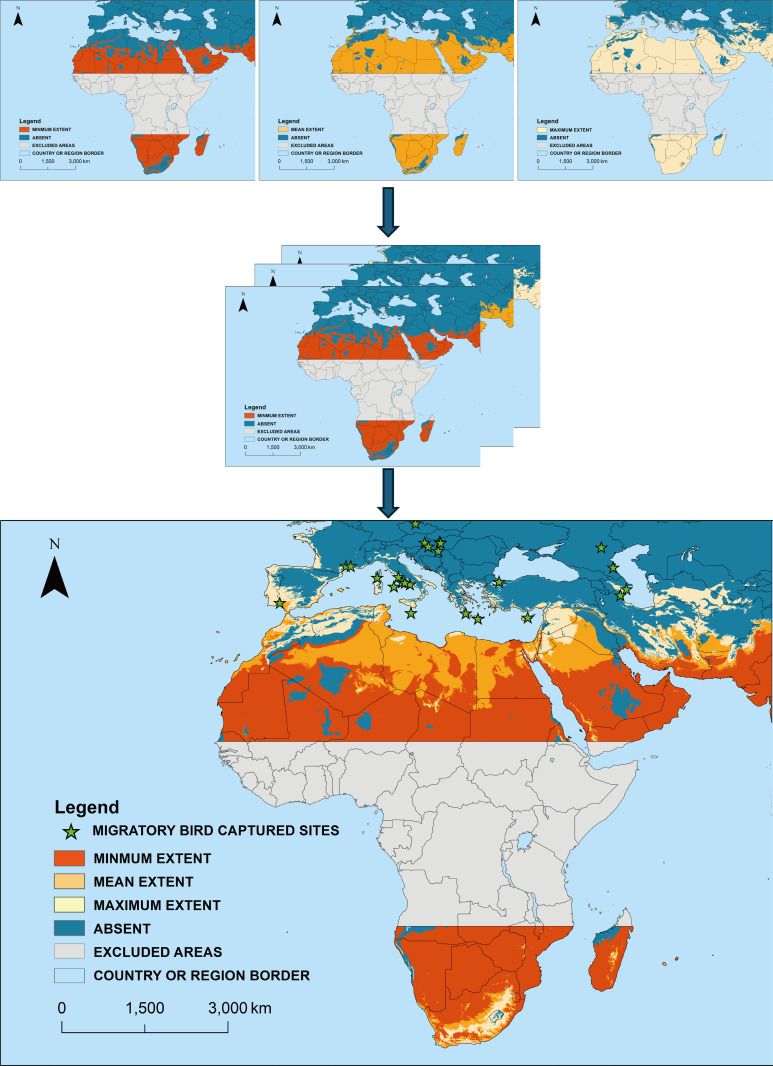
Potential suitable area for *H. rufipes* for the period 1990-2018. Excluded areas represent regions within 15° of the Equator, where minimal temperature fluctuations reduce the effectiveness of season detection based on average temperature. Base layers from (https://hub.arcgis.com/datasets/esri::world-countries-generalized/explore), Terms of use (https://www.esri.com/content/dam/esrisites/en-us/media/legal/product-specific-terms-of-use/e300.pdf).

Model 3, with the lowest omission rate, was used for binary transformation. The potential suitable areas for *H. rufipes* are displayed in Fig 2. Three estimates of predicted probability of presence were derived from the model replicates: the minimum, mean, and maximum values among 20 replicates. Each of these values were coded as present if the predicted probability of presence value exceeded threshold (10% training presence threshold =0.618), and as absent otherwise. Notably, the three categories represent the maximum, minimum, and average results from 20 replicates, which produced varying outcomes due to cross-validation, as each replicate used a different training set. For example, the red area (minimum extent) indicates the potential suitable area from the replicate with the smallest extent, while the orange area represents the average result from the 20 replicates. The light-yellow area indicates the results from the replicate with the greatest extent. We overlapped the three extents into one figure to illustrate the differences.

In the replicate with minimum extent, areas with a possibility of being suitable for *H. rufipes* include the Middle East, and Sub-Saharan Africa. With over 20 replicates, the potential suitable areas expanded to include Northern Africa, the southern coastal areas of Spain, Italy and Greece, and additional countries in the Middle East, such as Afghanistan and Iraq. In the replicate with maximum extent, these areas covered almost the entire African continent and extended into more territories in the Western and Southern Iberian Peninsula, the coastal regions of Italy, Greece, and Türkiye, as well as parts of Central Asia, including regions in Iran, Afghanistan, and surrounding areas. Notably, even the northern part of Italy and the south of Great Britain were predicted to be suitable for *H. rufipes* in the maximum extent replicate.

Additionally, migratory birds travel long distances twice annually, moving between breeding and non-breeding regions. These migrations, spanning both regional and intercontinental scales within a short period, allow immature stages of *Hyalomma rufipes* to hitch a ride on the birds. This association enables the ticks to cross geographical barriers such as seas and deserts, facilitating their widespread dispersal. We visually overlaid the sites where *H. rufipes* infested migratory birds were captured during migration on the model result map to better understand the tick invasion risk in a one health perspective (Fig 2). Capture sites allocated in coastal areas of southern Spain, France and Italy, Greece, Malta, Cyprus, Hungary, Czech, Russia, Azerbaijan, Türkiye.

## Discussion

Environmental niche modeling has been widely used to define the set of environmental variables that better define the regions colonized by arthropods vectors of pathogens affecting human and animal health [12,56–58]. The results provided useful information in vector surveillance and control programs. Some studies have specifically analyzed the potential suitable habitats of *H. marginatum*, the vector of CCHF, in certain areas [12,13,59]. However, for *H. rufipes*, another competent vector of CCHF, it is still unclear the distribution of its potential suitable habitat. Our study modeled the natural area of colonization with climate features for the period 1990 to 2018 consistent with the recording of most records of the tick. Unlike most studies rely on gridded “tailored” variables (commonly called “bio-variables”) obtained from sources like WorldClim or CHELSA, without any further consideration about its impact on the biology of modelled organism(s), our study utilized harmonic regression to convert climate data to a daily scale. This approach allowed us to generate variables based on their ecological relevance to the target species, overcoming the limitations in coverage that restrict both the study period and the choice of variables from existing datasets.

The results were converted to a binary format based on a 10% training presence threshold. These settings reflect a conservative and practical approach, avoiding the use of lower thresholds that might make the results appear more attractive but less reliable. It is probable that suitable areas for *H. rufipes* exist in Northern and Southern Africa, the Western and Southern Iberian Peninsula, most of the Arabian Peninsula, coastal areas of southern France and Italy, Cyprus, Türkiye, parts of Greece, the Caucasus region, Iran, Afghanistan, and their surrounding areas. *Hyalomma rufipes* is an Afrotropical species [60] and permanent populations have not been confirmed in Europe [61]. We displayed the results using three colors to advance interpretation the meaning comprehensively. Generally, the mean extent (orange + red) is the most convincing, practical, and universally applicable for developing surveillance strategies or tick control and eradication plans, as it represents the average of 20 replicates. However, for forward-looking studies such as invasion prevention, it is advisable to also consider the maximum extent. This larger extent is more proactive and preventive, as it was derived from a single replicate that identified the largest potential suitable area. It is reported that there have been human cases of CCHF in the Arabian Peninsula [14]. Study published by Jane exclude the vector role of *H. marginatum* in the region [62]. Given that the species of vectors shown to have the competence necessary for the maintenance and transmission of CCHFV are limited [6], we reasonably suspect that *H. rufipes* plays a significant role in disease transmission in the Arabian Peninsula.

*Hyalomma rufipes* is widespread in Southern Africa, but recent studies have pinpointed punctual records of the tick outside of its known distribution across Eurasia [22,23,25,27,63]. Specifically, in Hungary, the first recorded occurrence of *H. rufipes* involved two male ticks found on cows in 2011, with another male found on cattle in 2021 [64]. In Germany, the first record of *H. rufipes* was reported in 2015 [19], followed by the collection of eight adult *H. rufipes* from a horse in Germany [63]. In 2019, one *H. rufipes* was collected from a horse in the Czech Republic, marking the first record of this species in the country [65]. These findings suggest increased molting or survival rates of *H. rufipes* in these invaded sites. A study of the year 2022 further hypothesized and demonstrated the presence of an indigenous *H. rufipes* population in Hungary [66]. Although no additional evidence of a permanent *H. rufipes* population in Europe has been observed, these reports warrant more attention on the invasion of *H. rufipes.*

As a two-host tick, the larvae and nymphs of *H. rufipes* feed on small mammals, ground-dwelling birds, and reptiles, while adult ticks feed on ungulates such as goats, camels, horses, and cattle, and occasionally on humans. The spread of *H. rufipes* through migratory birds and the emergence of adults have been reported in several countries where the tick was previously considered non-endemic [25,67]. Invasive alien species often undergo several key processes as they establish and spread in new environments: Introduction, establishment and spread. By integrating data on migratory birds infested with *H. rufipes* with our findings, it becomes clear that the Mediterranean region warrants closer monitoring for tick surveillance and prevention. This includes actively sampling ticks from domestic animals, wildlife, and the environment to monitor species distribution, abundance, and seasonal patterns. Effective tick management also relies on the careful use of acaricides, with strategies to prevent resistance. Public education is essential, focusing on physical measures like protective clothing and tick repellents, as well as chemical methods such as sprays. Raising awareness about early detection and safe tick removal can help reduce the risk of tick-borne diseases and encourage community involvement in prevention efforts. Migratory birds facilitate the spread of *H. rufipes* to these areas, while these regions are also climatically suitable for the survival of *H. rufipes*. If other biotic factors, such as the availability of suitable hosts, are favorable, *H. rufipes* is likely to establish a permanent population in the southern coastal areas of Spain, Italy and Greece. Additionally, one CCHFV-positive *H. rufipes* nymph and one CCHFV-positive *H. rufipes* larva were collected from two migratory birds during the springs of 2017 and 2018, respectively, in Italy [17]. Migratory birds introduce not only the vector directly but also, indirectly, may introduce the CCHFV as infected ticks. Thus, active surveys in risky areas, where the tick is not yet established but the environmental condition supporting it survival, are advisable for rapid intervention and prevention. Considering the high-risk priority of CCHF by WHO, the active, supervised surveys of the tick should be part of the routine surveillance for vector borne diseases in any European territory.

During data collection and reporting processes, data are often recorded solely for the purpose of the original study, while other valuable information may be unconsciously overlooked. It represents a significant loss for potential reuse in future studies. Here we advocate for sharing morphological characteristics through photographs or molecular identification, and for recording and reporting coordinates with as much precision as possible (up to 100 meters resolution, which corresponds to approximately 3-4 decimal places for latitude and longitude), rather than relying on approximate district names, local administrative divisions, or other artifacts that may distort accurate reporting. In this sense, the compilation of large dataset of ticks and associated traits [67] should be considered for large regions of the world.

In this study, we focused on environmental variables to identify the potential suitable areas for *H. rufipes* using a well-known performing method like MaxEnt. As a result, the analysis does not account for factors such as host abundance, human disturbance, landscape features, or vegetation [51]. The presence data are limited by their availability in the literature and were not randomly sampled, which may introduce sampling bias. To mitigate this, we reduced spatial redundancy by removing sites within 1 km of each other, resulting in the exclusion of only 4 records. After assessing the impact of these removals, we chose to retain all the data to ensure a comprehensive representation of tick presence, as this did not affect the outcome. Another limitation of this study is the exclusion of areas within 15° north and south of the equator. This exclusion is due to minimal temperature fluctuations near the equator, which reduce the effectiveness of season detection methods based on temperature variation. Despite this, we believe our chosen variable set performs better in describing and projecting potential suitable areas for *H. rufipes* compared to other sets selected solely by statistical methods, because our variables were selected for their ecological relevance to *H. rufipes* [15] and are more up-to-date than others.

## Conclusion

This study explored the environmental niche of *H. rufipes*, a vector of CCHF, using MaxEnt modeling to predict potential suitable areas out of its natural occurrence area. These areas include regions in Northern and Southern Africa, the Western and Southern Iberian Peninsula, the Arabian Peninsula, southern coastal areas of France and Italy, Cyprus, Türkiye, parts of Greece, the Caucasus region, and Iran and Afghanistan. This research addresses a major knowledge gap regarding the potential distribution of *H. rufipes*. Additional data on migratory birds further supports the need for surveillance of *H. rufipes* in key areas, as migratory avians are introducing *H. rufipes* annually.

## Supporting information

S1 Table Presence sites of *Hyalomma rufipes .
* (XLSX)

S2 Table Migratory birds captured sites. (XLSX)

S3 Table Calibration results. (XLSX)

## References

[1] Bente DA, Forrester NL, Watts DM, McAuley AJ, Whitehouse CA, Bray M. Crimean-Congo hemorrhagic fever: history, epidemiology, pathogenesis, clinical syndrome and genetic diversity. Antiviral Res. 2013;100(1):159–89. doi: 10.1016/j.antiviral.2013.07.006 23906741

[2] Belobo JTE, Kenmoe S, Kengne-Nde C, Emoh CPD, Bowo-Ngandji A, Tchatchouang S, et al. Worldwide epidemiology of Crimean-Congo hemorrhagic fever virus in humans, ticks and other animal species, a systematic review and meta-analysis. PLoS Negl Trop Dis. 2021;15(4):e0009299. doi: 10.1371/journal.pntd.0009299 33886556 PMC8096040

[3] Fanelli A, Buonavoglia D. Risk of Crimean Congo haemorrhagic fever virus (CCHFV) introduction and spread in CCHF-free countries in southern and Western Europe: A semi-quantitative risk assessment. One Health. 2021;13:100290. doi: 10.1016/j.onehlt.2021.100290 34307823 PMC8283130

[4] Hawman DW, Feldmann H. Crimean-Congo haemorrhagic fever virus. Nat Rev Microbiol. 2023;21(7):463–77. doi: 10.1038/s41579-023-00871-9 36918725 PMC10013989

[5] Fillâtre P, Revest M, Tattevin P. Crimean-Congo hemorrhagic fever: An update. Med Mal Infect. 2019;49(8):574–85. doi: 10.1016/j.medmal.2019.09.005 31607406

[6] Gargili A, Estrada-Peña A, Spengler JR, Lukashev A, Nuttall PA, Bente DA. The role of ticks in the maintenance and transmission of Crimean-Congo hemorrhagic fever virus: A review of published field and laboratory studies. Antiviral Res. 2017;144:93–119. doi: 10.1016/j.antiviral.2017.05.010 28579441 PMC6047067

[7] Temur AI, Kuhn JH, Pecor DB, Apanaskevich DA, Keshtkar-Jahromi M. Epidemiology of Crimean-Congo Hemorrhagic Fever (CCHF) in Africa-underestimated for decades. Am J Trop Med Hyg. 2021;104(6):1978–90. doi: 10.4269/ajtmh.20-1413 33900999 PMC8176481

[8] Fereidouni M, Apanaskevich DA, Pecor DB, Pshenichnaya NY, Abuova GN, Tishkova FH, et al. Crimean-Congo hemorrhagic fever virus in Central, Eastern, and South-eastern Asia. Virol Sin. 2023;38(2):171–83. doi: 10.1016/j.virs.2023.01.001 36669701 PMC10926685

[9] Dowall SD, Carroll MW, Hewson R. Development of vaccines against Crimean-Congo haemorrhagic fever virus. Vaccine. 2017;35(44):6015–23. doi: 10.1016/j.vaccine.2017.05.031 28687403 PMC5637709

[10] Bonnet SI, Vourc’h G, Raffetin A, Falchi A, Figoni J, Fite J, et al. The control of *Hyalomma* ticks, vectors of the Crimean-Congo hemorrhagic fever virus: Where are we now and where are we going?. PLoS Negl Trop Dis. 2022;16(11):e0010846. doi: 10.1371/journal.pntd.0010846 36395110 PMC9671348

[11] Sorvillo TE, Rodriguez SE, Hudson P, Carey M, Rodriguez LL, Spiropoulou CF, et al. Towards a sustainable one health approach to crimean-congo hemorrhagic fever prevention: focus areas and gaps in knowledge. Trop Med Infect Dis. 2020;5(3):113. doi: 10.3390/tropicalmed5030113 32645889 PMC7558268

[12] Hekimoglu O, Elverici C, Kuyucu AC. Predicting climate-driven distribution shifts in *Hyalomma marginatum* (Ixodidae). Parasitology. 2023;150(10):883–93. doi: 10.1017/S0031182023000689 37519234 PMC10577666

[13] Estrada-Peña A, Sánchez N, Estrada-Sánchez A. An assessment of the distribution and spread of the tick *Hyalomma marginatum* in the western Palearctic under different climate scenarios. Vector Borne Zoonotic Dis. 2012;12(9):758–68. doi: 10.1089/vbz.2011.0771 22448680

[14] Perveen N, Khan G. Crimean-Congo hemorrhagic fever in the Arab world: A systematic review. Front Vet Sci. 2022;9:938601. doi: 10.3389/fvets.2022.938601 36176697 PMC9513365

[15] Estrada-Peña A. The climate niche of the invasive tick species *Hyalomma marginatum* and *Hyalomma rufipes* (Ixodidae) with recommendations for modeling exercises. Exp Appl Acarol. 2023;89(2):231–50. doi: 10.1007/s10493-023-00778-3 36881286 PMC10097758

[16] Schulz A, Barry Y, Stoek F, Pickin MJ, Ba A, Chitimia-Dobler L, et al. Detection of Crimean-Congo hemorrhagic fever virus in blood-fed *Hyalomma* ticks collected from Mauritanian livestock. Parasit Vectors. 2021;14(1):342. doi: 10.1186/s13071-021-04819-x 34187526 PMC8244218

[17] Mancuso E, Toma L, Pascucci I, d’Alessio SG, Marini V, Quaglia M, et al. Direct and indirect role of migratory birds in spreading CCHFV and WNV: A Multidisciplinary Study on three stop-over islands in Italy. Pathogens. 2022;11(9):1056. doi: 10.3390/pathogens11091056 36145488 PMC9505975

[18] Chen Z, Li Y, Liu Z, Yang J, Yin H. The life cycle of *Hyalomma rufipes* (Acari: Ixodidae) under laboratory conditions. Exp Appl Acarol. 2012;56(1):85–92. doi: 10.1007/s10493-011-9490-0 21913002

[19] Chitimia-Dobler L, Nava S, Bestehorn M, Dobler G, Wölfel S. First detection of *Hyalomma rufipes* in Germany. Ticks Tick Borne Dis. 2016;7(6):1135–8. doi: 10.1016/j.ttbdis.2016.08.008 27567111

[20] Song J, Li W, Wu Z, Xue Y, Pan Y, Shi W. Identification part of biologic characteristic research of *Hyalomma rufipes*. Chinese J Vet Sci Technol. 2001;31 29–30.

[21] Lang J, Shan Y, Zhang M, Liu J, Wang F. The complete mitochondrial genome of *Hyalomma rufipes* (Acari: Ixodidae) from China and comparative analysis of mitogenomes in genus *Hyalomma*. Int J Acarol. 2022;48(2):87–97. doi: 10.1080/01647954.2022.2030794

[22] Bakirci S, Sarali H, Aydin L, Latif A, Eren H, Karagenç T. *Hyalomma rufipes* (Koch, 1844) infesting cattle in the West Aegean region of Turkey Aegean region of Turkey. Turk J Vet Anim Sci. 2011; 35(5):359–63. doi: 10.3906/vet-0910-160

[23] Földvári G, Szabó É, Tóth GE, Lanszki Z, Zana B, Varga Z, et al. Emergence of *Hyalomma marginatum* and *Hyalomma rufipes* adults revealed by citizen science tick monitoring in Hungary. Transbound Emerg Dis. 2022;69(5):e2240–8. doi: 10.1111/tbed.14563 35436033 PMC9790508

[24] Chaligiannis I, Musella V, Rinaldi L, Cringoli G, de la Fuente J, Papa A, et al. Species diversity and spatial distribution of ixodid ticks on small ruminants in Greece. Parasitol Res. 2016;115(12):4673–80. doi: 10.1007/s00436-016-5259-z 27655133

[25] Grandi G, Chitimia-Dobler L, Choklikitumnuey P, Strube C, Springer A, Albihn A, et al. First records of adult *Hyalomma marginatum* and *H. rufipes* ticks (Acari: Ixodidae) in Sweden. Ticks Tick Borne Dis. 2020;11(3):101403. doi: 10.1016/j.ttbdis.2020.101403 32037097

[26] Springer A, Shuaib YA, Isaa MH, Ezz-Eldin MI-E, Osman AY, Yagoub IA, et al. Tick Fauna and Associated *Rickettsia*, *Theileria*, and *Babesia spp*. in Domestic Animals in Sudan (North Kordofan and Kassala States). Microorganisms. 2020;8(12):1969. doi: 10.3390/microorganisms8121969 33322349 PMC7763929

[27] Hansford KM, Carter D, Gillingham EL, Hernandez-Triana LM, Chamberlain J, Cull B, et al. *Hyalomma rufipes* on an untraveled horse: Is this the first evidence of *Hyalomma* nymphs successfully moulting in the United Kingdom?. Ticks Tick Borne Dis. 2019;10(3):704–8. doi: 10.1016/j.ttbdis.2019.03.003 30876825

[28] Kratou M, Belkahia H, Selmi R, Andolsi R, Dhibi M, Mhadhbi M, et al. Diversity and phylogeny of cattle ixodid ticks and associated spotted fever group *Rickettsia spp*. in Tunisia. Pathogens. 2023;12(4):552. doi: 10.3390/pathogens12040552 37111438 PMC10146803

[29] Andrade AFA de, Velazco SJE, De Marco Júnior P. ENMTML: An R package for a straightforward construction of complex ecological niche models. Environ Model Software. 2020;125:104615. doi: 10.1016/j.envsoft.2019.104615

[30] Escobar LE. Ecological niche modeling: an introduction for veterinarians and epidemiologists. Front Vet Sci. 2020;7:519059. doi: 10.3389/fvets.2020.519059 33195507 PMC7641643

[31] Cobos ME, Peterson AT, Barve N, Osorio-Olvera L. kuenm: an R package for detailed development of ecological niche models using Maxent. PeerJ. 2019;7:e6281. doi: 10.7717/peerj.6281 30755826 PMC6368831

[32] Feng X, Park DS, Walker C, Peterson AT, Merow C, Papeş M. A checklist for maximizing reproducibility of ecological niche models. Nat Ecol Evol. 2019;3(10):1382–95. doi: 10.1038/s41559-019-0972-5 31548646

[33] Merow C, Smith MJ, Silander JA Jr. A practical guide to MaxEnt for modeling species’ distributions: what it does, and why inputs and settings matter. Ecography. 2013;36(10):1058–69. doi: 10.1111/j.1600-0587.2013.07872.x

[34] Arguez A, Applequist S. A harmonic approach for calculating daily temperature normals constrained by homogenized monthly temperature normals. J Atmospheric and Oceanic Technol. 2013;30(7):1259–65. doi: 10.1175/jtech-d-12-00195.1

[35] Estrada-Peña A, Estrada-Sánchez A, Estrada-Sánchez D, de la Fuente J. Assessing the effects of variables and background selection on the capture of the tick climate niche. Int J Health Geogr. 2013;12:43. doi: 10.1186/1476-072X-12-43 24069960 PMC3849650

[36] Estrada-Peña A, Gray JS, Kahl O, Lane RS, Nijhof AM. Research on the ecology of ticks and tick-borne pathogens--methodological principles and caveats. Front Cell Infect Microbiol. 2013;3:29. doi: 10.3389/fcimb.2013.00029 23964348 PMC3737478

[37] Estrada-Peña A, Estrada-Sánchez A, de la Fuente J. A global set of Fourier-transformed remotely sensed covariates for the description of abiotic niche in epidemiological studies of tick vector species. Parasit Vectors. 2014;7:302. doi: 10.1186/1756-3305-7-302 24984933 PMC4089935

[38] Scharlemann JPW, Benz D, Hay SI, Purse BV, Tatem AJ, Wint GRW, et al. Global data for ecology and epidemiology: a novel algorithm for temporal Fourier processing MODIS data. PLoS One. 2008;3(1):e1408. doi: 10.1371/journal.pone.0001408 18183289 PMC2171368

[39] Robert J. Hijmans RB, Dyba K, Pebesma E, Sumner MD. terra: Spatial Data Analysis. R package version 1.7-78. 2024.

[40] R Core Team. R: A Language and Environment for Statistical Computing. R Foundation for Statistical, Computing:Vienna, Austria 2024.

[41] Kung-Sik Chan BR. TSA: Time Series Analysis. R package version 1.3.1. 2022.

[42] Killick R, Eckley IA. changepoint: AnRPackage for Changepoint Analysis. J Stat Soft. 2014;58(3):1-19. doi: 10.18637/jss.v058.i03

[43] Fiocruz/CAVAISC - Coleção de Artrópodes Vetores Ápteros de Importância em Saúde das Comunidades [Internet]. 2023 [cited 2023-09-06]. Available from: doi: 10.15468/vrioeo

[44] INSDC Host Organism Sequences [Internet]. 2023 [cited 2023-09-06]. Available from: doi: 10.15468/e97kmy

[45] INSDC Sequences [Internet]. 2023 [cited 2023-09-06]. Available from: doi: 10.15468/sbmztx

[46] LACM Entomology Collection [Internet]. 2023 [cited 2023-09-06]. Available from: doi: 10.15468/kc9hyp

[47] SysTax- Zoological Collections [Internet]. 2023 [cited 2023-09-06]. Available from: doi: 10.15468/zyqkbl

[48] International Barcode of Life project (iBOL) [Internet]. 2023 [cited 2023-09-06]. Available from: doi: 10.15468/inygc6

[49] Hadley Wickham RF, Lionel Henry, Kirill Müller, Davis Vaughan. dplyr: A Grammar of Data Manipulation. R package version 1.1.4. 2023.

[50] Owens HL, Campbell LP, Dornak LL, Saupe EE, Barve N, Soberón J, et al. Constraints on interpretation of ecological niche models by limited environmental ranges on calibration areas. Ecol Model. 2013;263:10–8. doi: 10.1016/j.ecolmodel.2013.04.011

[51] Barve N, Barve V, Jiménez-Valverde A, Lira-Noriega A, Maher SP, Peterson AT, et al. The crucial role of the accessible area in ecological niche modeling and species distribution modeling. Ecol Model. 2011;222(11):1810–9. doi: 10.1016/j.ecolmodel.2011.02.011

[52] Peterson AT, Papeş M, Soberón J. Rethinking receiver operating characteristic analysis applications in ecological niche modeling. Ecol Model. 2008;213(1):63–72. doi: 10.1016/j.ecolmodel.2007.11.008

[53] García-Callejas D, Araújo MB. The effects of model and data complexity on predictions from species distributions models. Ecol Model. 2016;326:4–12. doi: 10.1016/j.ecolmodel.2015.06.002

[54] Warren DL, Wright AN, Seifert SN, Shaffer HB. Incorporating model complexity and spatial sampling bias into ecological niche models of climate change risks faced by 90 California vertebrate species of concern. Divers Distrib. 2013;20(3):334–43. doi: 10.1111/ddi.12160

[55] ESRI 2016. ArcGIS Desktop: Release 10.5 Redlands, CA: Environmental Systems Research Institute.

[56] Clarke-Crespo E, Moreno-Arzate CN, López-González CA. Ecological niche models of four hard tick genera (Ixodidae) in Mexico. Animals (Basel). 2020;10(4):649. doi: 10.3390/ani10040649 32283708 PMC7222792

[57] Escobar LE, Romero-Alvarez D, Leon R, Lepe-Lopez MA, Craft ME, Borbor-Cordova MJ, et al. Declining prevalence of disease vectors under climate change. Sci Rep. 2016;6:39150. doi: 10.1038/srep39150 27982119 PMC5159793

[58] Aguilar-Domínguez M, Moo-Llanes DA, Sánchez-Montes S, Becker I, Feria-Arroyo TP, de León AP, et al. Potential distribution of Amblyomma mixtum (Koch, 1844) in climate change scenarios in the Americas. Ticks Tick Borne Dis. 2021;12(6):101812. doi: 10.1016/j.ttbdis.2021.101812 34416565

[59] Bah MT, Grosbois V, Stachurski F, Muñoz F, Duhayon M, Rakotoarivony I, et al. The Crimean-Congo haemorrhagic fever tick vector *Hyalomma marginatum* in the south of France: Modelling its distribution and determination of factors influencing its establishment in a newly invaded area. Transbound Emerg Dis. 2022;69(5):e2351–65. doi: 10.1111/tbed.14578 35511405 PMC9790221

[60] Apanaskevich DA, Horak IG. The genus *Hyalomma* Koch, 1844: v. re-evaluation of the taxonomic rank of taxa comprising the *H. (Euhyalomma) marginatum* koch complex of species (Acari: Ixodidae) with redescription of all parasitic stages and notes on biology. Int J Acarol. 2008;34(1):13–42. doi: 10.1080/01647950808683704

[61] Guglielmone AA, Nava S, Robbins RG. Geographic distribution of the hard ticks (Acari: Ixodida: Ixodidae) of the world by countries and territories. Zootaxa. 2023;5251(1):1–274. doi: 10.11646/zootaxa.5251.1.1 37044740

[62] Messina JP, Wint GRW. The spatial distribution of crimean-congo haemorrhagic fever and its potential vectors in Europe and Beyond. Insects. 2023;14(9):771. doi: 10.3390/insects14090771 37754739 PMC10532370

[63] Chitimia-Dobler L, Schaper S, Rieß R, Bitterwolf K, Frangoulidis D, Bestehorn M, et al. Imported *Hyalomma* ticks in Germany in 2018. Parasit Vectors. 2019;12(1):134. doi: 10.1186/s13071-019-3380-4 30909964 PMC6434826

[64] Hornok S, Horváth G. First report of adult *Hyalomma marginatum rufipes* (vector of Crimean-Congo haemorrhagic fever virus) on cattle under a continental climate in Hungary. Parasit Vectors. 2012;5:170. doi: 10.1186/1756-3305-5-170 22889105 PMC3436687

[65] Hubálek Z, Sedláček P, Estrada-Peña A, Vojtíšek J, Rudolf I. First record of *Hyalomma rufipes* in the Czech Republic, with a review of relevant cases in other parts of Europe. Ticks Tick Borne Dis. 2020;11(4):101421. doi: 10.1016/j.ttbdis.2020.101421 32360146

[66] Keve G, Csörgő T, Benke A, Huber A, Mórocz A, Németh Á, et al. Ornithological and molecular evidence of a reproducing *Hyalomma rufipes* population under continental climate in Europe. Front Vet Sci. 2023;10:1147186. doi: 10.3389/fvets.2023.1147186 37035818 PMC10073722

[67] Estrada-Peña A, de la Fuente J. Machine learning algorithms for the evaluation of risk by tick-borne pathogens in Europe. Ann Med. 2024;56(1):2405074. doi: 10.1080/07853890.2024.2405074 39348264 PMC11443563

